# Posterodorsal Medial Amygdala Mediates Tail‐Pinch Induced Food Intake in Female Rats

**DOI:** 10.1111/jne.12390

**Published:** 2016-05-03

**Authors:** M. H. Hu, Z. Bashir, X. F. Li, K. T. O'Byrne

**Affiliations:** ^1^ Division of Women's HealthFaculty of Life Sciences and MedicineKing's College LondonLondonUK

**Keywords:** amygdala, comfort eating, CRF, food intake, stress

## Abstract

Comfort eating during periods of stress is a common phenomenon observed in both animals and humans. However, the underlying mechanisms of stress‐induced food intake remain elusive. The amygdala plays a central role in higher‐order emotional processing and the posterodorsal subnucleus of the medial amygdala (MePD), in particular, is involved in food intake. Extra‐hypothalamic corticotrophin‐releasing factor (CRF) is well recognised for mediating behavioural responses to stress. To explore the possible role of amygdala CRF receptor activation in stress‐induced food intake, we evaluated whether a stressor such as tail‐pinch, which reliably induces food intake, would fail to do so in animals bearing bilateral neurotoxic lesions of the MePD. Our results showed that ibotenic acid induced lesions of the MePD markedly reduced tail‐pinch induced food intake in ovariectomised, 17β‐oestradiol replaced rats. In addition, intra‐MePD (right side only) administration of CRF (0.002 or 0.02 ng) via chronically implanted cannulae resulted in a dose‐dependent increase in food intake, although higher doses of 0.2 and 2 ng CRF had less effect, producing a bell shaped curve. Furthermore, intra‐MePD (bilateral) administration of the CRF receptor antagonist, astressin (0.3 μg per side) effectively blocked tail‐pinch induced food intake. These data suggest that the MePD is involved in stress‐induced food intake and that the amygdala CRF system may be a mediator of comfort eating.

Stress‐induced feeding or ‘comfort eating’ is a commonly experienced phenomenon and is characterised by an increase in food intake when faced with emotionally or physically challenging events 1, 2. It is a likely contributor to the increased prevalence of obesity in those suffering from stress‐related conditions. Studies have linked major depressive disorder and generalised anxiety disorder in adolescence to an increased risk of obesity in adulthood 3. Despite the commonality of this behaviour, the responsible mediators of stress‐induced feeding remain elusive.

Exposure to various psychological stressors, such as restraint and footshock, inhibits feeding behaviour in rodents 1, 4. By contrast, mild stressors such as tail‐pinch reliably induce food intake in rats 1, 4. The classical stress neuropeptide, corticotrophin‐releasing factor (CRF) was shown to have anorexic and orexigenic actions in the central nervous system. Intracerebroventricular administration of CRF suppressed food intake 5, 6, whereas the CRF receptor antagonists, α‐helical CRF_(9–41)_ and astressin‐B, significantly increased food intake 5 or augmented tail‐pinch induced food intake 7. Conversely, i.c.v. administration of CRF was also shown to increase food intake, an effect that was blocked by selective CRF type 1 receptor (CRF‐R1) antagonism 4. These results suggest that CRF is involved in food intake (both hyperphagia and anorexia) in a stressor‐dependent manner. However, the site of action and the detailed mechanism involved remain to be established.

Animal studies have demonstrated the activation of the extra‐hypothalamic CRF system in modulating food intake 6, 8, specifically during appetitive and aversive events 9. A dense concentration of CRF‐containing neurones and their receptors lies in the centromedial nuclear group of the amygdala 10, 11, 12. A general increase in CRF mRNA has been reported as a result of acute and chronic stress challenges, such as restraint stress or footshock, as well as corticosterone delivery in the amygdala, specifically in the central nucleus (CeA) 13. Moreover, microinjection of CRF‐R1 antagonist into the CeA completely blocks the excessive intake of palatable food, as well as anxiety‐like behaviour 14, suggesting that intra‐amygdala CRF mediates comfort eating during stress.

Within the amygdala, the highest levels of CRF‐R1 expression are evident in the cortical and medial (MeA) amygdaloid nuclei, with moderate levels of CRF‐R2 expression in the medial amygdala 15. Lesions of the amygdala have long been known to induce hyperphagia and obesity in cats, dogs and monkeys 16. More specifically, the posterodorsal subnucleus of the medial amygdala (MePD) has been determined as the effective site in rats, with lesions of this area disrupting normal feeding behaviour and resulting in hyperphagia and obesity 16. Activation in the MePD after restraint stress 17 or exposure to a predator 18 confirms its participation in psychological stress and is indicative of its functional relationship with anxiety or fear behaviour. However, there is little evidence for the role of the MePD in modulating stress‐induced food intake. In the present study, we test the hypothesis that the MePD mediates stress‐induced food intake involving local CRF signalling. We investigate whether bilateral neurotoxic lesions of the MePD block the mild stress of tail‐pinch induced food intake in ovariectomised 17β‐oestradiol replaced rats. We also investigate whether intra‐MePD administration of CRF *per se* alters food intake. Finally, we examine whether tail‐pinch induced food intake is blocked by intra‐MePD administration of the CRF‐R antagonist, astressin.

## Materials and methods

### Animals and surgical procedures

Adult female Sprague–Dawley rats (Harlan Laboratories Ltd., Bicester, UK) weighing 200–250 g were housed individually under a 12 : 12 h light/dark cycle (lights on 07.00 h) at 22 ± 2 °C and provided with standard rat chow and water *ad lib*. All surgical procedures were carried out under ketamine (100 mg/kg i.p.; Pharmacia and Upjohn, Crawley, UK) and Rompun (10 mg/kg i.p.; Bayer, Leverkusen, Germany) anaesthesia. Rats were bilaterally ovariectomised and implanted with a Silastic capsule (inner diameter 1.57 mm; outer diameter 3.18 mm; Sanitech, Havant, UK) filled to a length of 25 mm with 17β‐oestradiol (E_2_) dissolved at a concentration of 20 mg/ml arachis oil (Sigma‐Aldrich, Poole, UK), producing circulating concentration of E_2_ (approximately 38 ± 1.2 pg/ml) as described previously 19. All animal procedures were performed in accordance to the UK Home Office Regulations.

#### Bilateral lesions of the MePD

Animals received bilateral MePD excitotoxic lesions (n = 12) via bilateral injections of 0.5 μl of ibotenic acid (10 μg/μl in sterile 0.1 m PBS, pH 7.4; Sigma‐Aldrich) using a 1‐μl Hamilton injection syringe connected with a glass pipette 20 at the coordinates: 3.4 mm lateral, 3.3 mm posterior to bregma and 8.6 mm below the surface of the dura 21. The sham lesions (n = 8) were carried out using the same procedure but using sterile 0.1 m sodium phosphate buffer. MePD lesions were performed at the time of ovariectomy.

#### Bilateral MePD cannulae implantation

To assess the effect of intra‐MePD administration of CRF or CRF‐R antagonist on food intake and tail‐pinch induced food intake, respectively, a separate group of rats (n = 35) was implanted with bilateral guide cannulae (22‐gauge; Plastics One Inc., Roanoke, VA, USA) aimed towards the MePD (coordinates for implantation: −3.3 mm posterior to bregma, 3.4 mm lateral and 8.6 mm below the surface of the dura) 21. The guide cannula was anchored to the skull using dental cement (Associated Dental Products Ltd., Swindon, UK) 19. The guide cannulae were fitted with dummy cannulae to prevent obstruction when not in use. All brain cannulae were implanted at the time of ovariectomy. After surgery, all rats were housed individually and allowed to recover for 10 days.

### Experimental procedures

#### Effect of MePD lesion on tail‐pinch induced food intake

On the morning of experimentation, food was removed 1 h before the tail‐pinch procedure. The animals were then individually moved to a separate procedure room for the tail‐pinch test. Pinch pressure was applied to the third of the tail extending from the root via a cotton‐padded clip for 1 min. Rats did not vocalise during the application of tail‐pinch pressure 1, 4. After the tail clip was removed, the rats were provided with pre‐weighted standard rat chow pellets. Thirty minutes later, the remaining food was weighed. As a control for tail‐pinch, the sham‐lesioned rats were monitored for food intake over a 30‐min period, as described above, but in the absence of tail‐pinch. All experiments started between 09.00 and 10.00 h.

#### Effect of intra‐MePD injection of CRF on food intake

Rats were trained prior to the start of the experiment to accustom them to the intracerebral injection procedure. Twice a day (morning and afternoon) for 3 days, rats were handled by light hand restraint when the dummy cannulae were removed and reinserted. On the morning of experimentation, rats were moved to a procedure room and food was removed for 1 h prior to the injection procedure. Rats were then assigned in a cross‐over order to either treatment or control groups. Rats were given a unilateral (right side) intra‐MePD injection of CRF (Sigma‐Aldrich) at doses 0.002, 0.02, 0.2 or 2 ng in 300 nl of artificial cerebrospinal fluid (aCSF) or aCSF as a control (300 nl). An internal cannula with extension tubing, preloaded with CRF, was inserted into the guide cannula, extending 1.0 mm beyond the tip to reach the right MePD. The distal end of the extension tubing was connected to a 2‐μl syring (SGE Analytical Science, Milton Keynes, UK). The extension tubing was held outside the cage to allow the rat to move freely. The 300 nl of CRF solution or aCSF was delivered over 2 min and the injection internal cannula was kept in the guide cannula for another 1 min to prevent backflow. After the injection, pre‐weighted standard chow was placed inside the cage. Food intake over the next 30 min was then measured. Each animal was used up to three occasions with a different dose on each occasion and a 3‐day interval between treatments.

#### Effect of intra‐MePD injection of CRF‐R antagonist on tail‐pinch induced food intake

On completion of the CRF treatments described above, the same animals were used for the CRF antagonist studies after a delay of 5–6 days. A bilateral intra‐MePD injection of astressin (0.3 μg per side dissolved in 300 nl of aCSF; Sigma‐Aldrich) or aCSF (300 nl/side) was administered over 2 min as described above. Fifteen minutes after injection, the tail‐pinch procedure was carried out as described above. After the tail‐pinch, pre‐weighted standard chow was placed inside the cage and food intake over the next 30 min was measured. Control animals were given bilateral intra‐MePD injections of aCSF or astressin in the absence of tail‐pinch. A cross‐over design was used for these experiments with a 3‐day interval between treatments. All experiments started between 09.00 and 10.00 h after a 1‐h fast period.

### Brain collection and histology verification of lesion site and cannula position

Animals were killed by decapitation after experimentation. The brains were removed and snap frozen on dry ice, and then stored at −80 °C and coronally sectioned (30 μm) at a later date using a cryostat (Bright lnstrument Co. Luton Ltd, UK). To evaluate the lesion sites, every fourth section throughout the MePD region corresponding to bregma −2.80 to −3.60 mm 21 was mounted and stained with cresyl violet. Slides were then viewed under a light microscope and images were taken using a digital camera (Zeiss, Oberkochen, Germany) attached to the microscope. Neurones were identified by appearance and Nissl substance in the cytoplasm. The boundaries of the MePD and cannulae placement were determined using neuroanatomical landmarks in comparison with the rat brain atlas 21. Animals with lesion or cannulae outside of the desired structure were excluded from subsequent analysis.

### Statistical analysis

Comparisons between rats that received ibotenic acid and sham lesions, CRF or CRF antagonist administration and their vehicle controls in the MePD with respect to food intake were made using anova followed by multiple comparisons with Dunn's Test. Data are presented as the mean ± SEM and P* *<* *0.05 was considered statistically statistically significant.

## Results

### Lesion and cannula position in the MePD

**Figure 1:**
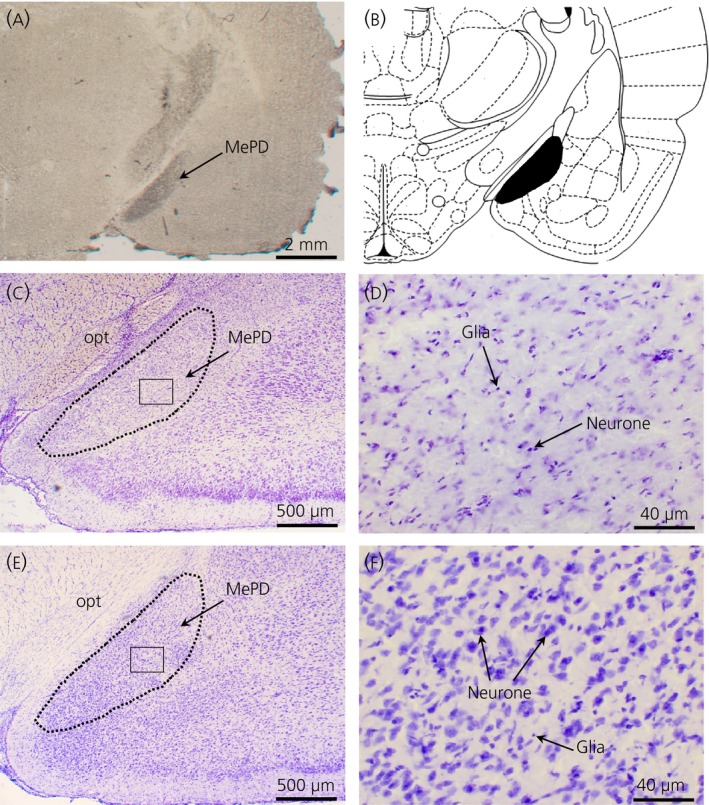
Coronal sections through the rat brain at the level of the posterodorsal medial amygdala (MePD) showing its spatial relation with the surrounding nuclei and ibotenate lesion, resulting in specific neuronal cell loss. (a) Section without staining showing the lesion in the MePD. (b) Schematic representation of site of lesion adapted from the rat brain atlas of Paxinos and Watson 21. Representative examples of a lesioned (c, d) and sham‐lesioned MePD (e, f), respectively. Opt, optic tract.

The extent and location of the lesions were confirmed by microscopic histological inspection, using cresyl‐violet staining. The presence of extensive neurone loss within the MePD was used as a parameter to determine the existence of significant lesions. Only bilateral lesions that destroyed the MePD at the same time as leaving the surrounding brain tissue largely intact were included in the present study (Fig. 1 a–d). Animals were excluded from the analysis as a result of unsuccessful lesioning, unilateral damage or damage extending outside of the MePD. Of the 12 rats that underwent neurotoxic lesioning, nine were confirmed with correct bilateral lesions in the MePD and included in the analysis. The remaining three rats with lesions outside of the MePD were excluded in the analysis. All vehicle‐injected rats sustained no obvious damage to the MePD (Fig. 1 e,f).

**Figure 2:**
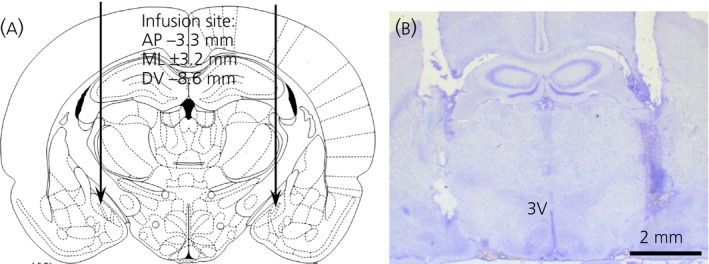
Schematic illustrations and photomicrograph of the cannulae target sites in the posterodorsal medial amygdala (MePD). (a) Schematic illustration showing the target site for bilateral cannulation of the MePD at bregma (AP) −3.3 mm, mediolateral (ML) ± 3.2 mm and dorsoventral (DV) −8.6 mm according to the rat brain atlas of Paxinos and Watson 21. Arrows point to the location of the cannulae tips. (b) Photomicrograph of a coronal brain section in a representative animal implanted with bilateral cannulae in the MePD. 3V, third ventricle.

Out of 35 rats, 29 rats with correct intra‐MePD placement of cannulae were confirmed by histological analysis with reference to the atlas of Paxinos and Watson 21 and included in the analysis. A representative example is shown in Fig. 2.

### Effect of MePD lesion on tail‐pinch induced food intake

**Figure 3:**
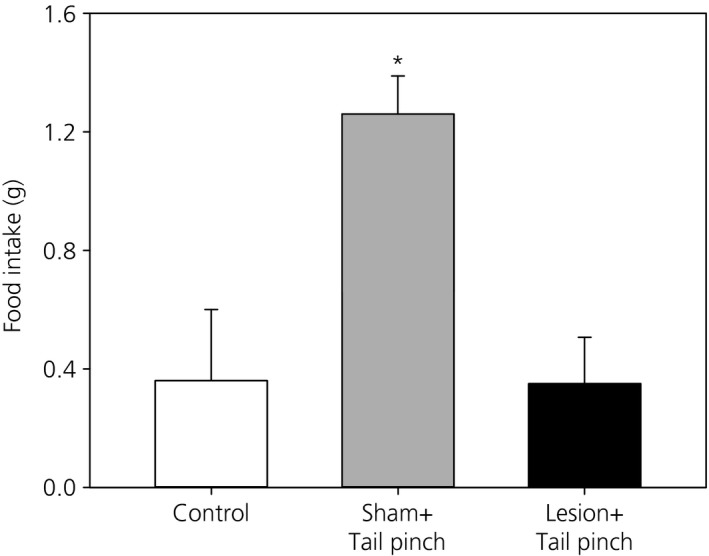
Effect of tail‐pinch induced stress on food intake in the posterodorsal medial amygdala (MePD) lesioned rats. Significantly reduced food intake is observed in MePD lesioned rats in comparison to the sham‐lesioned animals in response to tail‐pinch. *P < 0.05 versus absence of tail‐pinch in sham‐lesioned animals (control) or lesioned rats exposed to tail‐pinch. Results represent the mean ± SEM (n = 5–9 per group).

To determine whether the MePD is involved in stress‐induced food intake, we measured the amount of food intake within a 30‐min period immediately after tail‐pinch in female rats with MePD lesion compared to sham‐lesioned controls. The comparison revealed that rats with MePD lesion had significantly reduced food intake (mean ± SEM: 0.35 ± 0.16 g, n = 9 per group) compared to their sham‐lesioned controls (1.26 ± 0.13 g, n = 8 per group) (P < 0.05) (Fig. 3). As an additional control, sham‐lesioned rats were monitored for food intake in the absence of tail‐pinch (0.36 ± 0.24 g, n = 5) (Fig. 3). There was no significant difference in food intake after tail‐pinch between misplaced lesioned rats (1.23 ± 0.15 g, n = 3, P > 0.05) and sham‐lesioned controls.

### Effect of intra‐MePD administration of CRF on food intake

**Figure 4:**
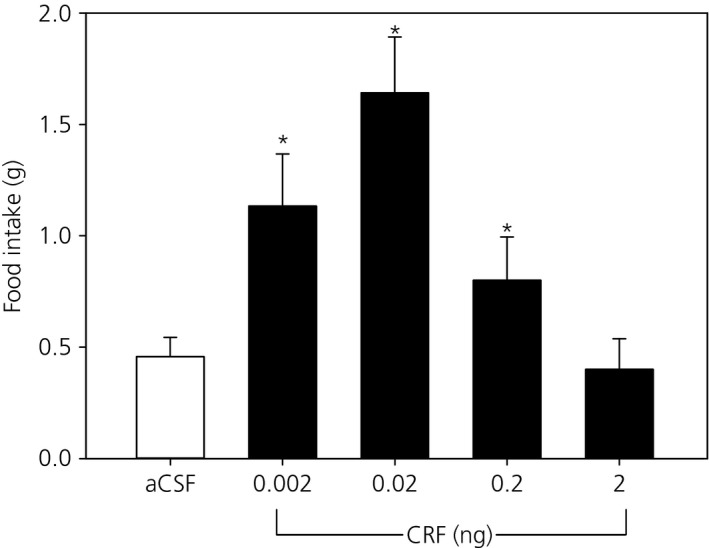
Effect of intra‐posterodorsal medial amygdala (MePD) microinjection of corticotrophin‐releasing factor (CRF) on food intake. A significant dose‐dependent increase in food intake at 30 min was observed at doses of 0.002 and 0.02 ng CRF in 300 nl of artificial cerebrospinal fluid (aCSF). However, food intake decreased at further higher doses providing a bell‐shaped dose response. *P < 0.05 versus controls. Results represent the mean ± SEM (n = 5–8 per group).

To determine whether CRF signalling in the MePD plays a role in food intake, CRF was micro‐infused into this brain area and food intake measured in the 30 min post‐treatment period. Compared with aCSF infusion (n = 5), unilateral intra‐MePD administration of CRF significantly increased food intake in a dose‐dependent manner (Fig. 4). A significant increase was observed at the dose of 0.002 ng (n = 7), with a maximum at 0.02 ng (n = 6) CRF compared to vehicle‐treated controls (P < 0.05) (Fig. 4). At higher doses of 0.2 ng (n = 8) and 2 ng (n = 6) CRF, food intake decreased towards control values, resulting in a bell‐shaped effect of CRF on food intake.

### Effect of intra‐MePD administration of CRF‐R antagonist on tail‐pinch induced food intake

**Figure 5:**
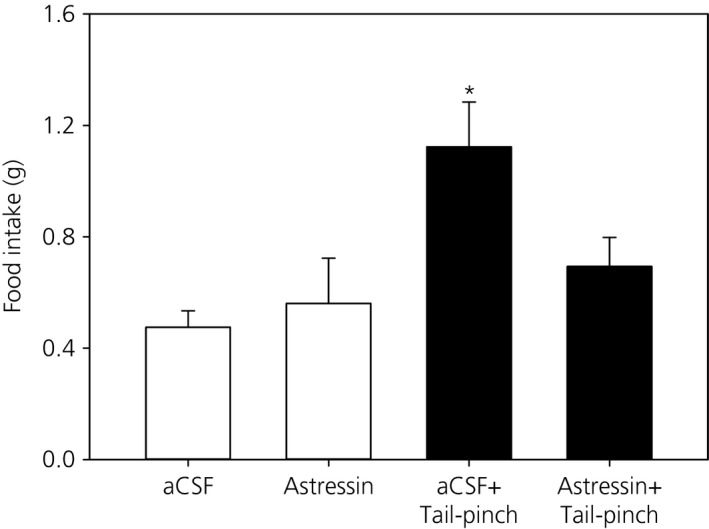
Effect of bilateral intra‐posterodorsal medial amygdala (MePD) microinjection of corticotrophin releasing factor receptor (CRF‐R) antagonist on tail‐pinch induced food intake. Acute 1‐min tail‐pinch significantly induced food intake that was blocked by microinjection of the nonselective CRF‐R antagonist, astressin, at a dose of 0.3 μg per side in 300 nl of artificial cerebrospinal fluid (aCSF) (n = 9 per group). Intra‐MePD administration of aCSF (n = 9) or astressin (n = 5) alone did not affect food intake. *P < 0.05 compared to other treatment groups.

As shown in Fig. 5, exposure to 1‐min tail‐pinch (n = 9) reliably increased food intake during the 30 min post‐stress period compared to aCSF‐treated controls (n = 9, P < 0.05). Tail‐pinch induced food intake was blocked by bilateral injection of the nonselective CRF antagonist astressin into the MePD (n = 9, P < 0.05). Intra‐MePD administration of astressin alone did not affect food intake (n = 5, P > 0.05).

## Discussion

In the present study, we have shown that lesions of the MePD caused a significant decrease in tail‐pinch induced food intake. Additionally, intra‐MePD administration of CRF resulted in a dose‐dependent response on food intake; significantly increasing food intake at lower doses but decreasing this response at higher doses to provide a bell‐shaped dose–response curve. Furthermore, intra‐MePD administration of the nonselective CRF‐R antagonist, astressin, significantly reduced tail‐pinch induced food intake. The MePD has previously been identified as a critical subnucleus of the amygdala in the regulation of food intake 22. Previous studies have shown that a substantial weight gain occurred only when the lesion extended into the MePD, and even a small lesion of the MePD resulted in hyperphagia and substantial weight gains 22, 23. The present study, however, reveals the first evidence for the involvement of CRF signalling in the MePD in stress‐induced feeding.

Although the role of the MePD in stress‐induced food intake has not been established, it has previously been shown that lesions of the MeA dampened stress‐induced increases in corticosterone release after 30 min of exposure to acute restraint stress 24, resulting in decreased food intake induced by serotonin agonism 25 and attenuated stress‐induced weight gain 24. The MeA serves as a key output nucleus of the amygdale complex, promoting HPA axis activation to acute psychogenic stress 26 and projecting to many brain regions, including the ventromedial nucleus of the hypothalamus (VMH) 27, 28, which is commonly associated with satiety. Lesions to the MeA result in a decreased number of CRF fibres in the VMH 29. In light of this, the findings of the present study suggest that CRF‐mediated stress‐induced feeding occurs along the MePD‐VMH pathway, although further research would be necessary to confirm this hypothesis.

Intra‐MePD administration of CRF resulted in a bell‐shaped effect on food intake, indicating that CRF has opposing effects depending on the dose, which is similar to previous studies using i.c.v. administration 4. In the latter study, CRF significantly increased food intake within 30 min after i.c.v. injection at doses of 2 and 10 ng but tended to decrease food intake at higher doses of 250 and 1250 ng in rats. Earlier studies demonstrated the anorexic effects of CRF on feeding behaviour when microgram doses were injected i.c.v. to fasted rats 30. These observations may be relevant to humans showing an increase in appetite with increasing stress but a gradual loss during periods of more severe stress 31, as well as in animal models using mild tail‐pinch stress, which increases food intake, whereas more severe stress such as restraint and footshock inhibit food intake 4, 32. Therefore, a differential role for endogenous CRF‐signalling in stress‐induced feeding is implicated; CRF alters food intake depending on the intensity of stress (i.e. mild versus severe). The findings of the present study suggest that food intake therefore may indicate the degree of stress severity.

The amygdala, especially its central nucleus, is a major course of CRF neuronal perikarya 29, 33 with a widespread projection, and is implicated in behavioural and physiological responses associated with fear, anxiety, stress, food intake and reward. However, whether these CRF neurones project to the MePD remains to be established. Bilateral intra‐MePD administration of astressin, a nonselective CRF‐R antagonist, significantly reduced tail‐pinch induced food intake. The orexigenic effect of tail‐pinch, as seen in the present study, is a robust observation 1, 4, 6, 7 and represents a reliable model of stress‐induced hyperphagia that may involve activation of the CRF system 5. However, previous investigations on the influence of brain CRF signalling pathway in the tail‐pinch feeding response have led to contradictory results 4, 5, 7. Treatment with a low dose (1 μg) of the CRF antagonist (i.c.v.) increased the duration of eating and amount of food intake during a 5‐min tail‐pinch session 5, 7. However, i.c.v. administration of higher doses of α‐helical CRF_(9–41)_ (5 and 25 μg), or a selective type 1 CRF‐R antagonist, CRA 1000, reduced or blocked tail‐pinch induced food intake 4. These discrepancies could be explained by differences in the experimental setting, such as rats being exposed to tail‐pinch for 5 min versus 1 min, feeding behaviour being evaluated only during the 5‐min tail‐pinch period instead of 30 min after tail‐pinch, and the different doses of CRF antagonist used. Moreover, different CRF‐R antagonists, such as α‐helical CRF_(9–41)_ and astressin‐B (i.e. a nonselective CRF‐R antagonist versus a selective CRF‐R1 antagonist), have been used in these studies. Both CRF‐R1 and ‐R2 are involved in the inhibition of food intake 34, 35, 36, whereas knockout of type 2 CRF‐R causes a ‘pro‐satiation’ effect after acute restraint stress 36. However, a differential role for either receptor subtype in food regulation remains uncertain. Within the amygdala, the highest levels of CRF‐R1 mRNA expression were evident in the medial nucleus, although moderate levels of CRF‐R2 were also detected in this same subnucleus 15. Because tail‐pinch induced food intake was blocked by intra‐MePD administration of astressin in the present study, as well as by i.c.v. administration of the CRF‐R1 selective antagonist 4, it would appear that tail‐pinch induced food intake behaviour may be mediated through CRF‐R1 in the MePD. However, further investigations into a possible differential role of type 1 and type 2 CRF‐R in MePD‐mediated food intake are needed.

In humans, comfort eating is characterised by ‘eating to feel better’, inferring that eating goes beyond the realm of meeting metabolic and energy needs alone, which begs the question of its evolutionary significance. Stimulation of reward pathways in the limbic system has been found, likely contributing to the elevation in mood after eating under stressful conditions 37. Studies in rodents have demonstrated attenuated stress responses (i.e. dampened HPA axis activation) to chronic restraint stress when given lard or sucrose compared to rodents given no food 38. This might suggest that comfort eating is a source of ‘self‐medication’ for the regulation of stress responses 39. However, although the tail‐pinch procedure readily increases feeding in this rodent model of acute mild stress, increased feeding in humans is generally associated with chronic stress and therefore a note of caution regarding any clear translational link is warranted.

Collectively, these findings provide new evidence for a role of medial amygdaloid CRF mediating stress‐induced feeding. This potentially provides a target brain area for treatment of obesity and related stress conditions.
